# Psychometric properties of the Vertigo symptom scale – Short form

**DOI:** 10.1186/1472-6815-8-2

**Published:** 2008-03-27

**Authors:** Kjersti Wilhelmsen, Liv Inger Strand, Stein Helge G Nordahl, Geir Egil Eide, Anne Elisabeth Ljunggren

**Affiliations:** 1 Department of Public Health and Primary Health Care, Section for Physiotherapy Science, University of Bergen, Norway; 2 National Centre for Vestibular Disorders, Department of Otorhinolaryngology/Head and Neck Surgery, Haukeland University Hospital, Bergen, Norway; 3 Department of Physical Therapy, Haukeland University Hospital, Bergen, Norway; 4 Centre for Clinical Research, Haukeland University Hospital, and Department of Public Health and Primary Health Care, University of Bergen, Norway

## Abstract

**Background:**

The aim of the study was to examine the psychometric properties of the Vertigo symptom scale – short form (VSS-SF), a condition-specific measure of dizziness, following translation of the scale into Norwegian.

**Methods:**

A cross-sectional survey design was used to examine the factor structure, internal consistency and discriminative ability (sample I, n = 503). A cross-sectional pre-intervention design was used to examine the construct validity (sample II, n = 36) of the measure and a test-retest design was used to examine reliability (sub-sample of sample II, n = 28).

**Results:**

The scree plot indicated a two factor structure accounting respectively for 41% and 12% of the variance prior to rotation. The factors were related to vertigo-balance (VSS-V) and autonomic-anxiety (VSS-A). Twelve of the items loaded clearly on either of the two dimensions, while three items cross-loaded. Internal consistency of the VSS-SF was high (alpha = 0.90). Construct validity was indicated by correlation between path length registered by platform posturography and the VSS-V (r = 0.52), but not with the VSS-A. The ability to discriminate between dizzy and not dizzy patients was excellent for the VSS-SF and sub-dimension VSS-V (area under the curve 0.87 and 0.91, respectively), and acceptable for the sub-dimension VSS-A (area under the curve 0.77). High test-retest reliability was demonstrated (ICC VSS-SF: 0.88, VSS-V: 0.90, VSS-A: 0.90) and no systematic change was observed in the scores from test to retest after 2 days.

**Conclusion:**

Using a Norwegian translated version of the VSS-SF, this is the first study to provide evidence of the construct validity of this instrument demonstrating a stable two factor structure of the scale, and the identified sub-dimensions of dizziness were related to vertigo-balance and autonomic-anxiety, respectively. Evidence regarding a physical construct underlying the vertigo-balance sub-scale was provided. Satisfactory internal consistency was indicated, and the discriminative ability of the instruments was demonstrated. The instrument showed satisfactory test-retest reliability.

## Background

Dizziness and balance problems, commonly seen in patients suffering from vestibular and related disorders, are inter-related phenomena denoting sensory dysfunctions [1-3]. Due to its subjective nature, dizziness remains a continuous clinical challenge. It has been sub-classified into *vertigo *– a false sensation of movement of self or environment, *presyncope *– sensations of light-headedness and impending fainting, *disequilibrium *– a sensation of imbalance and/or postural instability, and *"other types of dizziness" *– a vague and floating sensation often accompanied by somatic symptoms [1]. This classification was introduced 30 years ago, and is still in use [1,2]. The nature of the symptoms is central for understanding the individual patient's condition and perceived sensation [4].

The Vertigo symptom scale (VSS), consisting of 36 items, addresses frequency and severity of dizziness symptoms within the last 12 months. Development of the VSS was based on interviews with patients experiencing dizziness/vertigo and on literature [5]. Two main dimensions were derived by principal component analysis (PCA) identifying vertigo-balance and autonomic-anxiety symptoms. Acceptable validity [6,7] and test-retest reliability [5] have been demonstrated for the long version of the scale. Modest correlation between the two sub-scales has been reported [5]. The scale discriminated between patients and healthy individuals [6], and the sub-scales discriminated between vertigo-balance and autonomic- anxiety dimensions in patients with dizziness [5,6]. Also, the autonomic-anxiety sub-scale of the VSS correlated with objective measures of psycho-physiological arousal in patients with vestibular disorders [7].

A shortened version of the scale (VSS-SF) comprising items extracted from the original scale [8], was introduced for use in clinical trials as a measure of symptom severity within the past month [9]. The VSS-SF consists of 15 items. Each item is scored on a 5-point scale (range 0–4), and a measure of symptom severity is obtained by summing the item scores. The total scale score ranges 0–60, higher scores indicating more severe problems. Severe dizziness has been defined as ≥ 12 points on the total scale [10]. The scale is suggested to comprise two sub-scales: 8 items relating to vertigo-balance (VSS-V, score ranging 0–32), and 7 items relating to autonomic-anxiety symptoms (VSS-A, score ranging 0–28) [9].

The VSS-SF has shown satisfactory internal consistency [11] and moderate test-retest reliability [12]. Other psychometric properties have to our knowledge not been examined. Considering the subjective nature of dizziness, self-report instruments capturing these symptoms may be valuable, provided that the measurement properties are sound. After translating the VSS-SF into Norwegian, the aims of the present study were to examine the factor structure, internal consistency, construct validity and the discriminative ability of the instrument, as well as its test-retest reliability.

## Methods

### Translation

The VSS-SF was translated from English into Norwegian through a process of review and modification recommended by the World Health Organisation [13]. The Norwegian version is presented along with the English version in the Appendix. Permission was given by Lucy Yardley to name it VSS-SF, NV (Norwegian Version). Separate translations were made by two physiotherapists familiar with dizzy patients and knowledgeable in English. Back-translation was performed by a bilingual person fluent in Norwegian at a professional level and with English as a native language. Slight modifications were made in items 3, 6 and 15 by deleting "feeling sick" and "swimmy". All data were collected using the translated version of the scale. For readability, the scale is referred to by its original name.

### Participants

All patients (denoted samples I and II), recruited from a balance clinic located in a tertiary hospital in Norway, were examined for complaints of persistent dizziness by an ear – nose – and throat specialist. The medical examination was supplemented by laboratory tests including static stabilometric testing of balance (Cosmogamma^©^, Bologna, Italy) registering postural sway as path lengths (in mm). Centre of pressure was sensed by three mechanical-electrical transducers (strain gauges) and relayed to a computer (12 bit A/D resolution and 10 Hz sampling frequency). A standardized test protocol was used [14].

#### Sample I

Patients seen in the balance clinic between 1992 and 2001 and diagnosed within vestibular and non-vestibular categories were identified, age ranging 18–70 years. Patients with dizziness of neurological or traumatological aetiologies were excluded. A total of 820 patients were invited to take part in a postal survey in 2002 comprising several questionnaires including the VSS-SF. The response rate was 67% (n = 549). Some forms (n = 86) had inadequate scoring. Imputation was possible in 40 of these (forms lacking one or two items only on the respective sub-scales). The remaining forms were discarded. The final sample included 503 patients.

#### Sample II

Another group of patients with persistent dizziness were invited to take part in a program of vestibular rehabilitation following the standard medical and laboratory examination. Patients were included if history suggested uncompensated vestibular function as a consequence of vestibular neuronitis. In the study period 36 patients fulfilled the inclusion criteria. A comprehensive test battery incorporating the VSS-SF was used prior to intervention. Testing was done within one month following the medical examination. For the test-retest study, patients were asked to complete the VSS-SF twice, 48 hours apart and return the form by mail.

As part of a larger study, examination of measurement properties of the VSS-SF was approved by the Regional Committee for Medical Research Ethics in Western Norway. Written informed consent was obtained from all the patients.

### Statistical analysis

Demographic and test data of the two samples were examined by descriptive statistics. Distribution of scores was examined by q-q plots and by comparing mean and median scores of the scale and subscales. As normality could be assumed, parametric statistics were used. Repeated measures and independent samples t-tests were used for examination of differences in scores. Statistical significance was set at p ≤ 0.05.

The underlying factor structure of the VSS-SF has not previously been examined although results from PCA using varimax rotation on the mother version of the scale are available [5,6]. As PCA does not take latent variables into consideration, the approach is not recommended for exploration of factor structures [15] and exploratory factor analysis (EFA) was therefore used to examine the instrument's underlying structure (sample I) [15]. Maximum likelihood parameter extraction and the subjective scree test were used to determine the number of factors to be retained for further analysis. The factor structure was then identified by using the method of oblimin rotation of the factors with delta = 0 [15] allowing for a moderate correlation. The stability of the identified dimensions over varying levels of correlation between the latent factors was then studied by varying the value of delta.

Internal consistency of the VSS-SF, VSS-V and VSS-A sub-scales was examined (sample I). Cronbach's alpha values ≥ 0.70 were considered satisfactory high [16]. Construct validity of the VSS-V was examined (sample II) by 1) correlating the sub-scales and balance as registered by posturography, and 2) correlating the two sub-scales of the VSS-SF, using Pearson's correlation (r) [16].

The scales' ability to discriminate between dizzy and not dizzy patients was examined (sample I) by Receiver Operating Characteristic (ROC) curves using 1) "still dizzy" and 2) vestibular/non-vestibular categories as dichotomous, dependent variables. The VSS-SF and sub-scales were used as independent variables. Split half techniques were used dividing the sample randomly into two groups (Group 1: "dizzy" n = 189, "not dizzy" n = 58. Group 2: "dizzy" n = 176, "not dizzy" n = 71) to examine the stability of the results. The area under the ROC curve (AUC) expresses discriminative ability: 0.7 ≤ AUC ≤ 0.8 is considered acceptable, 0.8 < AUC ≤ 0.9 excellent, and AUC > 0.9 outstanding [17]. As a screening instrument, sensitivity (correctly classifying the dizzy individuals) should be maximized and specificity (correctly classifying the not-dizzy individuals) optimized [18]. Cut-off points for obtaining the best discriminative ability of the total scale and sub-scales were examined.

Test-retest reliability was explored in a sub-sample (n = 28) of sample II, and reported by intraclass correlation (ICC) coefficients [19]. Values ≥ 0.70 were considered satisfactory [18]. SPSS version 14 for Windows was used to analyze data.

## Results

Descriptive information of the samples is given in Table 1. Most participants in both samples were women. In sample I, vestibular diagnoses was the most common and represented by Ménière's disease, vestibular schwannoma, benign paroxysmal positional vertigo and vestibular neuritis sequele. The non-vestibular category comprised non-otogenic and cervicogenic dizziness. An average of 56 months (standard deviation (SD): 30) had passed since the medical examination, but the majority reported dizziness with recent dizzy spells at the time of the survey. In sample II, the majority of patients were classified as having peripheral vestibular disorders, and all were dizzy at the time.

**Table 1:** Demographic data of samples

Variable	Sample I n = 503	Sample II n = 36
Women; n (%)	303 (60)	22 (61)
Age; mean year (SD), min-max	50.0 (11.7), 18–71	48.4 (11.6), 24–73
Vestibular diagnosis; n (%)	311 (62)	26 (72)
Still dizzy; n (%)	365 (73)	36 (100)
Spells of dizziness within the last month; n (%)	295 (59)	36 (100)
Dizziness duration; mean month (SD), min-max	104.1 (79.0), 8–636	31.3 (50.0), 1–234

SD: standard deviation

**Figure 1:**
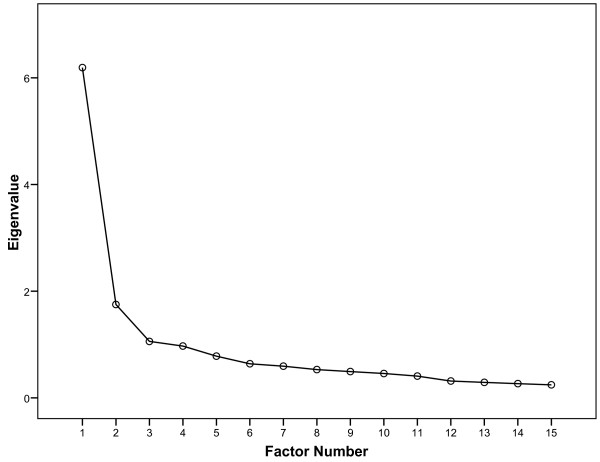
**Scree plot**. Scree plot for determination of number of factors retained for rotation.

The EFA revealed three factors with eigenvalues greater than 1 that explained 60% of the variance prior to rotation. The first factor explained 41% while the second explained 12%. A third factor which explained 7% of the variance comprised items related to time on the balance dimension (e.g. dizziness of short or long duration). The scree plot (Figure 1) indicated two factors to be retained for rotation. Results of the rotation showed that 12 of the items clearly loaded on one of the factors only, seven items on factor 1, corresponding to the VSS-V sub-scale, while five items loaded on factor 2, the VSS-A sub-scale (Table 2). The remaining 3 items, e.g. items 3 (nausea), 7 (headache, or feeling pressure in the head) and item 12 (feeling faint, about to black out), loaded on both factors (Table 2). The correlation between the factors was moderate (0.56) with delta set at zero.

**Table 2:** Comparison of factor values. Comparison between factor values on the Vertigo symptom scale – short form in the Norwegian sample by exploratory factor analysis, and the values from similar items by PCA in the full scale (Yardley et al, 1999)^a^

	Norwegian sample^b ^n = 509		Mexican sample n = 172		UK hospital sample n = 127		UK primary care sample n= 143	
	Balance	Anxiety	Balance	Anxiety	Balance	Anxiety	Balance	Anxiety

4. A feeling that either you, or things around you, are spinning or moving, lasting more than 20 minutes	0.84	-0.18	0.59^d^	-0.09	0.71^d^	-0.08	0.64^d^	-0.08
6. A feeling of being dizzy, disoriented or [or "swimmy]^c^, lasting all day	0.81	-0.10	0.71^d^	0.08	0.73^d^	0.04	0.69^d^	0.12
10. Feeling unsteady, about to loose balance, lasting more than 20 minutes	0.80	-0.01	0.69^d^	-0.12	0.77^d^	-0.03	0.68^d^	0.08
8. Unable to stand or walk properly without support, veering or staggering to one side	0.67	0.07	0.40	0.38	0.35	0.27	0.35	0.54
1. A feeling that either you, or things around you, are spinning or moving, lasting less than 20 minutes	0.61	0.09	0.53^e^	0.12	0.71^e^	-0.13	0.44^e^	0.14
15. A feeling of being dizzy disoriented [or "swimmy"]^c^, lasting less than 20 minutes	0.60	0.10	0.46^e^	0.30	0.70^e^	0.17	0.53^e^	0.11
13. Feeling unsteady, about to loose balance, lasting less than 20 minutes	0.58	0.14	0.62^e^	0.28	0.71^e^	0.13	0.58^e^	0.10
11. Excessive sweating	0.09	0.82	0.19	0.19	0.12	0.47	0.18	0.60
2. Hot or cold spells	-0.02	0.81	0.25	0.67	0.16	0.59	0.12	0.65
5. Heart pounding or fluttering	-0.04	0.56	0.23	0.58	0.02	0.66	0.09	0.64
9. Difficulty breathing, been short of breath	0.02	0.55	0.08	0.62	0.06	0.53	-0.02	0.72
14. Pains in the heart or chest region	0.05	0.45	0.09	0.67	-0.14	0.52	-0.10	0.52
12. Feeling faint, about to black out	0.43	0.32	0.26	0.02	0.30	0.45	0.06	0.60
3. Nausea [feeling sick]^1^, vomiting	0.35	0.31	0.35	0.58	0.36	0.40	0.23	0.56
7. Headache, or feeling of pressure in the head	0.33	0.33	0.23	0.71	0.06	0.52	0.11	0.58

^a ^Yardley et al: **Relationship between physical and psychosocial dysfunction in Mexican patients with vertigo: a cross-cultural validation of the Vertigo symptom scale, ***J Psychosom Res *1999, 46: 63–74.

^b ^Exploratory factor analysis with maximum likelihood extraction, oblimin rotation with delta = 0.

^c ^Not included in the Norwegian version.

^d ^Compared with the category "several hours" in the original version.

^e ^Compared with the category "2–20 minutes" in the original version

Cronbach's alpha, indicating internal consistency was 0.90 for the VSS-SF, 0.88 for the VSS-V and 0.81 for the VSS-A. Construct validity was indicated as the correlation value between the VSS-V sub-scale and path length was moderate (r = 0.52, p < 0.01) while it was low and not significant between the VSS-A sub-scale and path length (r = - 0.19, p = 0.30). Low and not significant correlation was also seen between the two sub-scales (r = 0.15, p = 0.41).

**Figure 2:**
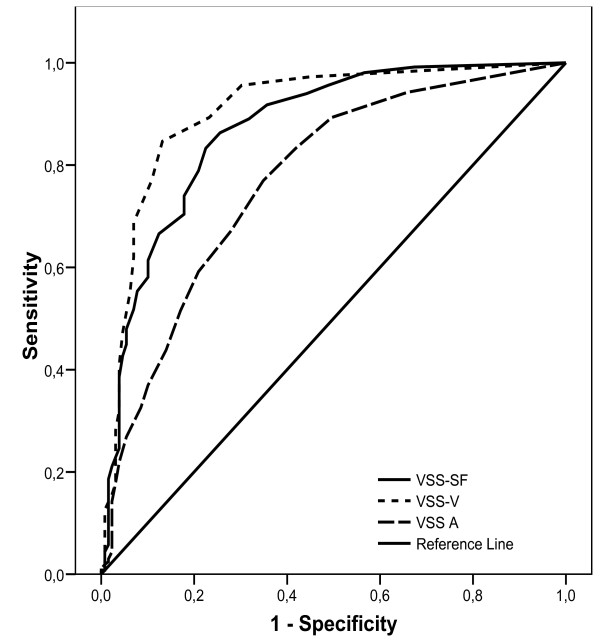
**Receiver operating characteristic curve**. Receiver operating characterstic (ROC) curve illustrating the ability of the Vertigo symptom scale-short form and sub-scales (VSS-V, VSS-A) to discriminate the "dizzy" from the "not dizzy" patients (N = 459).

In sample I, more severe symptoms were reported by the currently dizzy than the not dizzy group (Table 3). The scales' ability to discriminate between the dizzy and not dizzy was satisfactory as indicated by the area under the ROC curve (Figure 2, Table 3). The cut-off values of 6.5 on the VSS-SF, 2.5 on the VSS-V, and 3.5 on the VSS-A had all acceptable sensitivity and specificity (Table 3). Stability of the scales' discriminative ability was demonstrated by similar results in the randomly established groups (split half techniques) as in the whole sample I. The instrument did not discriminate between dizziness of vestibular and non-vestibular origin.

**Table 3:** Symptom scores. The degree of dizziness symptoms registered by the Vertigo symptom scale-short form and reported by dizzy (n = 365) and not dizzy (n = 129) responder in sample I, and the scales' ability to discriminate between dizzy and not dizzy responders

	Groups	Symptom score	Area under the curve	95% CI	Cut-off value	Sensitivity	Specificity
VSS-SF	Dizzy	17.2 (10.1)^a^					
	Not dizzy	5.0 (7.3)					
			0.87	(0.83, 0.91)	6.5	0.86	0.73
VSS-V	Dizzy	9.6 (6.5)^a^					
	Not dizzy	1.8 (3.8)					
			0.91	(0.87, 0.94)	2.5	0.89	0.77
VSS-A	Dizzy	7.6 (5.2)^a^					
	Not dizzy	3.2 (4.1)					
			0.77	(0.72, 0.82)	3.5	0.77	0.65

SD: standard deviation, CI: Confidence interval.

^a^p < 0.01 by independent samples t-test.

VSS-SF: Vertigo symptom scale – short form,

VSS-V: Vertigo-balance sub-scale,

VSS-A: Autonomic-anxiety sub-scale

**Table 4:** Test-retest scores. Test-retest of symptom scores and reliability of the Vertigo symptom scale-short Form as indicated by intraclass correlation coefficient (ICC) (n = 28)

Scale (range)	Test Mean (SD)	Re-test Mean (SD)	ICC (95% CI)
VSS-SF (0–60)	14.3 (7.7)	13.7 (8.6)	0.88 (0.77, 0.94)
VSS-V (0–32)	8.6 (6.0)	8.9 (6.8)	0.90 (0.80, 0.95)
VSS-A (0–28)^a^	5.7 (4.2)	4.8 (4.5)	0.90 (0.79, 0.95)

SD: standard deviation, CI: confidence interval.

VSS-SF: Vertigo symptom scale – short form,

VSS-V: Vertigo-balance sub-scale,

VSS-A: Autonomic-anxiety sub-scale.

^a^p < 0.01 by repeated measures t-test.

The sub-sample from sample II participating in the test-retest reliability study, consisted of 61% women, mean age; 47.9 years. The mean duration of dizziness was 30 months. At test, all items on the VSS-SF were scored by all patients. At retest imputation of scores was done in two forms (one item score lacking on each). The results are presented in Table 4. Test-retest reliability was highly satisfactory; ICC values ranging 0.88 to 0.90 for the total and the sub-scales. No change in scores was seen from test to retest.

## Discussion

This study complements previous examination of measurement properties of the original short-form instrument. Reliability and validity have scarcely been addressed, and the underlying dimensions have not been systematically explored in previous studies. It is also the first report of a translation of the VSS-SF into Norwegian, performed according to recommended guidelines [13]. The semantic and technical equivalence of items was kept as close as possible to the original. Apart from the two words deleted, the remaining English words and concepts were considered relevant and adequate for dizzy patients in the Norwegian culture. The modifications did not appear to alter the meaning of the questions. The translated version was pilot tested in a few dizzy patients (n = 4), finding no need for change of wording. Response categories and scoring system were kept the same as in the original scale. Problems with the response categories have previously been reported [20], as patients systematically left out answers of 0 (never), if non-symptomatic. No particular difficulties were met when using the Norwegian version of the VSS-SF in the present study.

The instrument has been suggested to comprise two dimensions of dizziness; a vertigo-balance and autonomic-anxiety dimension [9]. A two-factor structure was supported by the present study, and the factors indicated a balance and an anxiety dimension. No definite rules for cut-off values of factor loadings exist, but loadings above 0.4 [6] and 0.5 [5] have been considered appropriate in previous studies of the VSS. Others have suggested that loadings between 0.2 and 0.4 may be sufficient, although modest [15]. Using factor loading values of at least 0.4 as cut-off, 12 of the items in the present study loaded satisfactorily, while the loading of three items (item 3, 7, 12) may be considered modest. As these items also cross-loaded, further exploration of the factor structure was carried out by altering the value of delta in the oblimin rotation method, however, the same structure and similar loading values were seen. A set-up with the results from the present study is presented along with the results from previous studies that used PCA (Table 2) [6], even if a direct comparison is not possible because of methodological differences between PCA and EFA. Cross-loading of item 3 is seen across all the four studies (Table 2) and high face validity for item 3 on the vertigo-balance dimension has previously been argued [6]. Item 7, loaded equally on both dimensions in the present study, contrary to what is seen in the other studies where it loaded clearly on the anxiety dimension (Table 2). Item 12 was also found to cross-load in the present study fairly similar to that of the UK hospital sample, while it loaded weakly on the balance dimension in the Mexican sample and clearly on the anxiety sub-scale in the UK primary care sample [6]. It is not uncommon to find items that cross-load [15], and overlap in symptomatology related to vertigo-balance and autonomic-anxiety may account for this cross-loading [5]. In spite of the cross-loading of some factors, the constellation of factors founded on the empirical distinction between the dimensions, allows construction of a two-factor scale covering vertigo-balance and autonomic anxiety dimensions of dizziness as originally suggested [9].

Internal consistency of the VSS-SF was satisfactory high, similar to what was reported by Yardley et al. [12] and somewhat higher than what was reported by Soderman et al. [11]. Internal consistency of the sub-scales, not previously examined, was also satisfactory, the alpha values being within recommended limits [16].

In discussion of construct validity, the various aspects of validity must be taken into consideration [21]. Development of the original long version of the scale was based on interviews, literature and on PCA which revealed two core sub-scales [5]. In the present study, a similar structure was found, e.g. a vertigo-balance and an autonomic-anxiety dimension. The sub-scales complement each other, and the total instrument covers a relative broad concept of dizziness. The autonomic-anxiety sub-scale of the long version has previously been shown to correlate with objective measures of psycho-physiological arousal [7], but the vertigo-balance sub-scale has not been explored along similar lines. Maintenance of balance, achieved by a process of active movements around the point of gravity [22] can be registered by posturography, and it is considered a gold standard in measuring balance. This biophysical measure was used to examine the construct underlying the VSS-V sub-scale. The combined findings of (moderate) correlation between the VSS-V sub-scale and path length, and lack of correlation between the VSS-A sub-scale and path length in the present study, support our assumption that the sub-scales measure somewhat different constructs, further supported by the lack of correlation between the two sub-scales [23]. Thus, there is support that the VSS-V sub-scale reflects a construct related to physical aspects of dizziness. The idea that self-perceived dizziness can reflect different constructs is of interest when it comes to rehabilitation. Identifying the type of dizziness experienced by the individual may result in more customized rehabilitation programs.

The scale discriminated in a stable manner between patients with and without persistent dizziness. In particular, the VSS-V sub-scale showed excellent discriminative ability while it was acceptable for the autonomic-anxiety scale. The latter sub-scale's discriminate ability might have been even better if we had been able to distinguish between groups with and without somatic anxiety, but data for this purpose was not available in the present study. Ability to discriminate between healthy and patients has been established in the long version of the scale [6,7], but not explored by ROC curve analysis. The established cut-off values may be useful in identifying patients in need of vestibular rehabilitation [18].

Test-retest reliability was satisfactory high on all scales. Satisfactory test-retest reliability is important when it comes to evaluating results from interventions. The reliability was almost as high as that reported on the sub-scales of the long version (VSS-V: r = 0.94, VSS-A: r = 0.95) [5]. The somewhat higher values seen for the long version of the instrument [5], may be due to a shorter test-retest interval (24 hour) and suggest a recall-bias, or to the fact that the long version is a somewhat broader measure of the construct of dizziness. The moderate long term (6 months) test-retest reliability (r = 0.60) previously reported for the short version [12], may be due to an actual change in the condition even if the patients had had dizziness of long duration. There are no definite guidelines as to how long the time interval should be in test-retest studies. However, it should be long enough to secure that previous self-reported responses are forgotten and short enough for the condition to remain stable [16]. A risk of recall bias within 48 hours may be argued. As the form was part of an extensive test battery, this risk was considered minimal. The sample consisted of patients with dizziness of relative long duration, implying that the possibility of a change during a period of 48 hours was small.

Examination of reliability should preferably be done with varying scale scores among the target population. In the present sample, scores extended from the lower to the middle range which is typical for patients with dizziness of long duration [10,23]. For samples of patients with chronic dizziness, test-retest reliability of the VSS-SF is satisfactory. As far as we know the scale has not been used systematically with patients in the acute condition, but our clinical experience has shown scores in the upper range of the scale. The scale has a potential to register symptoms in the acute condition, but the rapid, spontaneous compensation taking place must be taken into consideration and the time period altered accordingly.

## Conclusion

The VSS-SF translated into Norwegian demonstrated satisfactory psychometric properties, many aspects being examined for the first time. The factor structure demonstrated two dimensions within the overall construct of dizziness, vertigo-balance and autonomic-anxiety dimensions and the vertigo-balance sub-scale was shown to capture a physical construct of dizziness. Satisfactory high internal consistency was demonstrated for the total and sub-scales. The instrument was able to distinguish between individuals with and without dizziness. Satisfactory test-retest reliability was demonstrated for the total and sub-scales. Measurement properties of the scale should also be tested in patients with acute vestibular dizziness.

## Abbreviations

VSS = Vertigo symptom scale, VSS-SF = Vertigo symptom scale-short form, VSS-V = vertigo-balance sub-scale, VSS-A = vertigo-anxiety sub-scale, EFA = exploratory factor analysis, PCA = principal component analysis, ROC = receiver operating characteristic curve, AUC = area under the (ROC) curve.

## Competing interests

The author(s) declare that they have no competing interest.

## Authors' contributions

KW designed the studies, collected data, performed statistical analysis and drafted the manuscript. LIS participated in designing the study, contributed in the analysis of data and drafting of the manuscript. SHGN and AEL both have contributed to the design of the studies and have assisted in drafting the manuscript. GEE has participated in the statistical analysis. All authors have read and approved the final manuscript.

## Appendix

See Table 5.

**Table 5:** Appendix.

**VSS-SF**	**VSS-SF, Norwegian version**
*How often in the past month have you had the following symptoms:*	*Hvor ofte har du i løpet av den siste måneden hatt følgende symptomer:*
1. A feeling that either you, or things around you, are spinning or moving, lasting less than 20 minutes	1. Følelsen av at du selv eller omgivelsene går rundt eller er i bevegelse, følelsen varer mindre enn 20 minutter
2. Hot or cold spells	2. Følt deg vekselvis varm eller kald
3. Nausea (feeling sick), vomiting	3. Kvalme, kastet opp
4. A feeling that either you, or things around you, are spinning or moving, lasting more than 20 minutes	4. Følelsen av at du selv eller omgivelsene går rundt eller er i bevegelse, følelsen varer over 20 minutter
5. Heart pounding or fluttering	5. Hjertebank eller flaksing
6. A feeling of being dizzy, disoriented or "swimmy", lasting all day	6. En følelse av å være svimmel eller desorientert og følelsen varer hele dagen
7. Headache, or feeling of pressure in the head	7. Hodepine eller en følelse av trykk i hodet
8. Unable to stand or walk properly without support, veering or staggering to one side	8. Ute av stand til å stå og gå uten støtte, går ustødig og trekker mot en side når du går
9. Difficulty breathing, been short of breath	9. Pustevansker, vært kortpustet
10. Feeling unsteady, about to loose balance, lasting more than 20 minutes	10. En følelse av å være ustø, at du holder på å miste balansen, og følelsen varer over 20 min.
11. Excessive sweating	11. Svettet veldig mye
12. Feeling faint, about to black out	12. Følelse av at du holder på å besvime
13. Feeling unsteady, about to loose balance, lasting less than 20 minutes	13. En følelse av å være ustø, at du holder på å miste balansen, og følelsen varer mindre enn 20 min.
14. Pains in the heart or chest region	14. Smerter i hjerte/brystområde
15. A feeling of being dizzy, disoriented or "swimmy", lasting less than 20 minutes	15. En følelse av å være svimmel eller desorientert, følelsen varer mindre enn 20 minutter
	
**Response categories**	
0 Never	0 Aldri
1 A few times	1 Noen ganger
2 Several times	2 Flere ganger
3 Quite often (every week)	3 Ganske ofte (hver uke)
4 Very often (most days)	4 Veldig ofte (nesten hver dag)
	
**VSS-V items**: 1, 3, 4, 6, 8, 10, 13, 15	
**VSS-A items**: 2, 5, 7, 9, 11, 12, 14	

## Pre-publication history

The pre-publication history for this paper can be accessed here:


